# Thiamine and diabetes: back to the future?

**DOI:** 10.1007/s00592-021-01752-4

**Published:** 2021-06-05

**Authors:** Elena Beltramo, Aurora Mazzeo, Massimo Porta

**Affiliations:** Dept. Medical Sciences, University of Torino, Corso AM Dogliotti 14, 10126 Torino, Italy

**Keywords:** Thiamine, Benfotiamine, Diabetes, Diabetic retinopathy, Diabetic nephropathy, Diabetic neuropathy

## Abstract

The first reports of a link between thiamine and diabetes date back to the 1940s. Some years later, a role for thiamine deficiency in diabetic neuropathy became evident, and some pilot studies evaluated the putative effects of thiamine supplementation. However, the administration of thiamine and its lipophilic derivative benfotiamine for the treatment of this complication gained consensus only at the end of the ‘90 s. The first evidence of the beneficial effects of thiamine on microvascular cells involved in diabetic complications dates to 1996: from then on, several papers based on *in vitro* and animal models have addressed the potential use of this vitamin in counteracting diabetic microangiopathy. A few pilot studies in humans reported beneficial effects of thiamine administration on diabetic nephropathy, but, despite all promising proofs-of-concept, the possible role of thiamine in counteracting development or progression of retinopathy has not been addressed until now. Thiamine is a water-soluble vitamin, rapidly expelled from the body, with no issues of over-dosage or accumulation; unfortunately, it is non-patentable, and neither industry nor independent donors are interested in investing in large-scale randomized controlled clinical trials to investigate its potential in diabetes and its complications. Consequently, science will not be able to disprove a promising hypothesis and, more importantly, diabetic people remain deprived of a possible way to ameliorate their condition.

*“I think that experiments with vitamins, which can at least do no harm, ought to be performed here in order to ascertain if a deficiency of the latter is not the real primary cause of the disease” Funk C, 1922 *[1]

*“In clinical trials of vitamin B1 (thiamin) in cases of diabetes the author, during the past few years, has found that administration of thiamin, either parenterally or orally, effects some improvement in the carbohydrate tolerance of the diabetic patient and has further observed an apparent economy of insulin in several instances after its administration” Bose JP, 1949 *[2]

## The first vitamin

Thiamine (vitamin B1) is a water-soluble vitamin belonging to the B group, the first to be isolated in 1926 [3]. As all water-soluble vitamins, it is rapidly expelled through the urinary system, does not accumulate in the body, and has no toxic effects. Nevertheless, it is an essential cofactor of glucose metabolism in almost all living organisms and a modulator of neuronal and neuro-muscular transmission in vertebrates [4]. Small amounts of it need to be assumed frequently with the diet [5]. Thiamine deficiency causes severe disorders, especially linked to the nervous system (*beriberi*, *Wernicke-Korsakoff syndrome*) [3, 6], which were once very frequent in people on poor, unvaried diets, lacking in whole grains, meat, legumes, and nuts, and easily resolved by thiamine assumption. Alcoholics are often thiamine deficient, mainly because of the impairing effects of chronic alcohol intake on intestinal absorptive mechanisms [7]. Low thiamine levels are often associated also with diabetes [8].

**Fig. 1:**
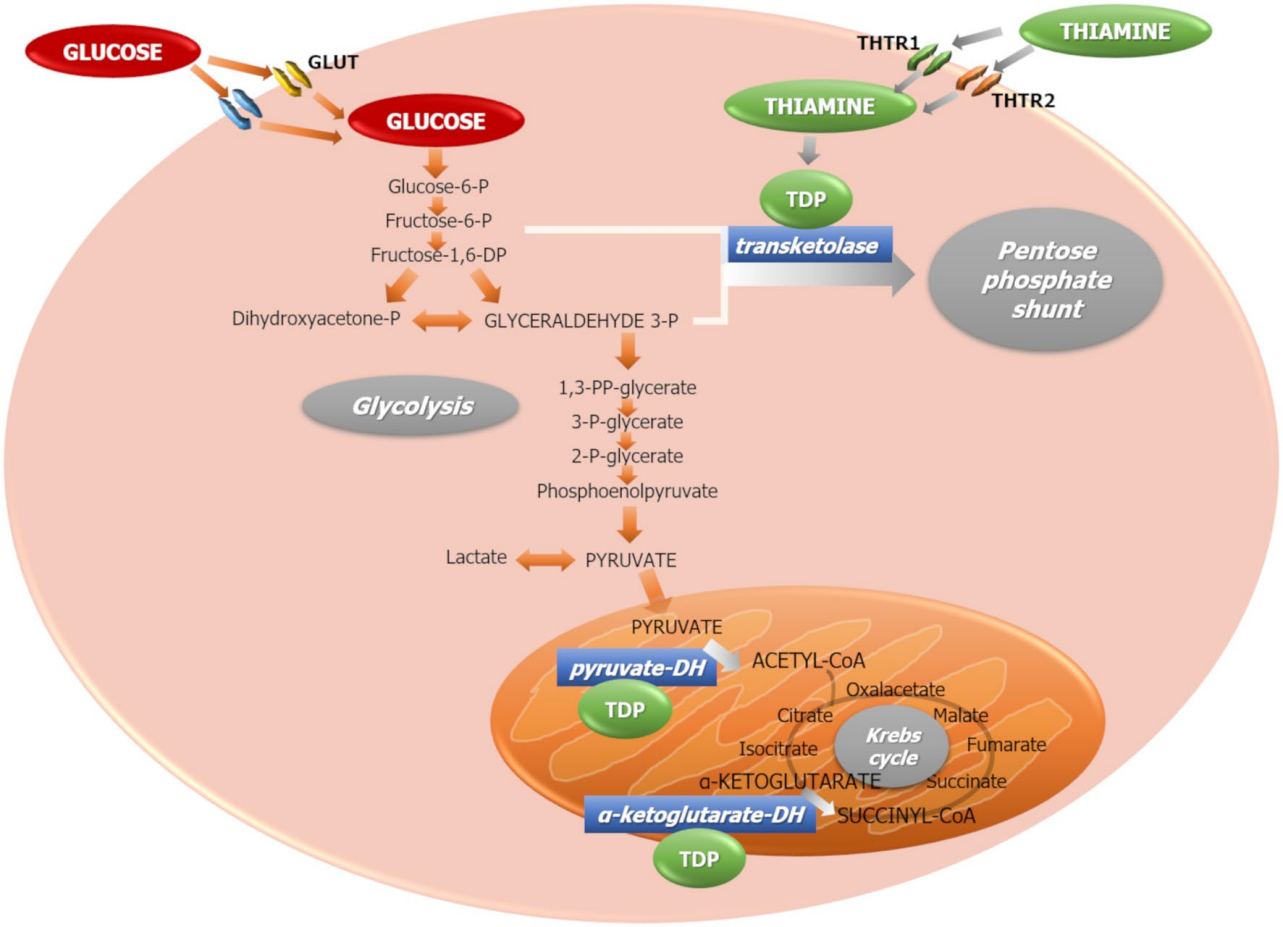
Thiamine enters the cells via thiamine transporters 1 (THTR1) and 2 (THTR2). Inside the cytoplasm, it is phosphorylated to its active form, thiamine diphosphate (TDP), an essential cofactor for several enzymes involved in glycolysis and Krebs cycle. Particularly important are transketolase, pyruvate-dehydrogenase and α-ketoglutarate-dehydrogenase. GLUT: glucose transporters

Absorption of thiamine is mainly mediated by two highly specific transporters, THTR1 and THTR2, largely present in almost all tissues [9]. At high concentrations, chiefly after pharmacological assumption, passive diffusion may also occur [10]. Once inside the cell, thiamine is phosphorylated to its active form, thiamine diphosphate (TDP), a cofactor for several enzymes involved mainly in glucose metabolism. These include: (1) transketolase (TK), which shifts excess glyceraldehyde-3-phosphate and fructose-6-phosphate from glycolysis toward the pentose phosphate shunt, thus eliminating highly reactive toxic metabolites from the cytoplasm; (2) the pyruvate-dehydrogenase complex, responsible for oxidative decarboxylation of pyruvate, the final product of glycolysis, to acetyl-CoA, which then enters the Krebs cycle; and (3) α-keto-glutarate-dehydrogenase, which facilitates the flow through the Krebs cycle (Fig. 1).

Genetic defects in intracellular thiamine transport are responsible for severe systemic disorders. Thiamine-responsive megaloblastic anemia (TRMA), a rare syndrome characterized by diabetes, anemia, and sensorineural deafness was described in 1978 [11], and later associated with a defect in the *SLC19A2* gene encoding for thiamine transporter-1 (THTR1) [12]. Since THTR1 is the only transporter of thiamine into pancreatic *β*-cells, cochlear cells, hemopoietic tissues and retinal pigment epithelial cells, its deficiency leads to cell apoptosis and organ failure [10]. Mutations in the *SLC19A3* gene encoding for thiamine transporter-2 (THTR2) are responsible for biotin-responsive basal ganglial disease [13] and thiamine-responsive encephalopathy [14].

A group of lipid-soluble thiamine derivatives, with higher absorption and bioavailability than water-soluble thiamine salts, was isolated in the 1960s [15]. Among these, one of the most effective is benfotiamine, which was specifically developed to improve bioavailability for oral administration [16, 17], and is currently marketed in some countries as an over-the-counter drug for the treatment of diabetic neuropathy.

## A brief history of thiamine and diabetes

A research on PubMed using the MeSH terms “thiamine” AND “diabetes” permits to date back to 1947 the first official report of a link between this vitamin and diabetes: that paper described thiamine deficiency in diabetic as compared to healthy rats [18]. However, proof of this association had been already published previously, though not listed in PubMed: two studies in 1940 described deficiency of cocarboxylase (i.e., thiamine diphosphate, TDP) in diabetic patients [19, 20], and were confirmed by subsequent observations [21]. In 1948, a British medical officer stationed in India reported on the reduction in glycemia and glycosuria in a group of diabetic subjects treated with 50 mg of daily intramuscular thiamine chloride for 10–14 days; thiamine administration also reduced the need for insulin in insulin-treated patients [2]. One year later, Markees and Meyer [22] reported on the beneficial effects of the administration of cocarboxylase in diabetic coma, and a debate on this topic arose in the following decade. Several subsequent reports postulated a synergic action of insulin and cocarboxylase in the treatment of diabetic coma and indicated as a putative mechanism of action the reduction of pyruvic acid, increased in decompensated diabetic patients [23–26]. The positive effects of thiamine and its phosphorylated forms on glycemia and glycosuria, already reported by Bose in 1948, were confirmed in the ‘50 s [27, 28]. Those reports leant toward a stronger effect of cocarboxylase rather than thiamine itself. However, soon after, administration of thiamine hydrochloride gained major credit because, while TDP cannot enter the cells directly, the free form of thiamine is recognized by its transporters and then phosphorylated to active TDP only inside the cytoplasm [26].

Unfortunately, after this rise of interest in the ‘50s, the potential of thiamine and its derivatives in the treatment of diabetes started to lose appeal. More than thirty years later, a new study evidenced thiamine deficiency in diabetic subjects [29], and confirming reports were published in the subsequent decades [30–33]. But, after the pioneering studies of the ‘50s, no further attempts to ameliorate the diabetic condition through the administration of thiamine were described until the dawn of the new millennium, when some studies reported on small groups of patients supplemented with vitamin B1 or one of its derivatives (benfotiamine, benzoyloxymethyl-thiamine). Valerio et al. [34] found no differences in HbA1c in type 1 diabetic children after 3-month treatment with benzoyloxymethyl-thiamine, while a group of type 2 diabetic patients experienced improved endothelial function and reduced development and progression of atherosclerosis after intravenous injection of thiamine [35]. Benfotiamine was shown to prevent macro- and micro-vascular endothelial dysfunction and oxidative stress following a meal rich in advanced glycation end products [36], and to ameliorate erythrocyte transketolase activity in type 2 diabetic patients [31]. One-month thiamine administration decreased glycemia and leptin concentrations in subjects with type 2 diabetes [37], and improvements were described in glucose tolerance [38] and blood pressure [39] in subjects with hyperglycaemia, following high-dose thiamine supplementation. Gestational diabetes was also associated with thiamine mishandling [40]: in these women, thiamine supplementation reduced inflammatory and oxidative markers [41]. Unfortunately, these timid approaches were never followed by proper randomized controlled clinical trials (RCTs).

## Thiamine and diabetic neuropathy

The literature is more abundant about thiamine treatment of diabetic neuropathy. The symptoms of a neurological and cardiovascular disease, later identified as *beriberi*, had been described in traditional Chinese medicine as far as the third century AD [42]. In 1897, the Dutch physician Christiaan Eijkman, sent to the Dutch East Indies to study *beriberi*, first linked this disorder to a poor diet (namely the assumption of polished rice) [43] and a few years later, in 1906, Hopkins hypothesized that food contains additional essential nutrients, apart from proteins, fat and carbohydrates [44]. Funk called these nutrients “vitamines”, after coming to the conclusion that they should belong to the amine group and be necessary for life (the “e” was dropped when it was discovered that not all the vitamins were effectively *amines*) [1]. Eijkman and Hopkins were awarded the Nobel Prize in Physiology or Medicine in 1929 for their discoveries, which led to the isolation of thiamine by Jansen and Donath in 1926, and the association of *beriberi* with thiamine deficiency [3]. Lack of thiamine is also the cause of the *Wernicke-Korsakoff syndrome*, or cerebral *beriberi*, a neuro-psychiatric disease described at the end of the nineteenth century and characterized by paralysis of eye movements, abnormal stance and gait, and deranged mental function [6]. Both syndromes are readily resolved by the administration of thiamine. Therefore, it was soon hypothesized that thiamine could be of help also for the treatment of diabetic neuropathy: the first hypothesis of such indication dates back to 1954 [45]. Eight years later, a review by Whitsell attests that thiamine chloride (as well as other vitamins belonging to the B group) has “been generously used in treatment [of diabetic neuropathy] with great enthusiasm in many reports” [46]. However, it was at the end of the ‘90s that the administration of thiamine and its lipophilic derivative benfotiamine in the treatment of this complication gained some consensus. Several subsequent reports confirmed that neuropathic symptoms and deficits, and nerve conduction velocity, were significantly improved by thiamine/benfotiamine administration in subjects with diabetic neuropathy [47–53].

## Thiamine and the microvascular complications of diabetes

**Fig. 2:**
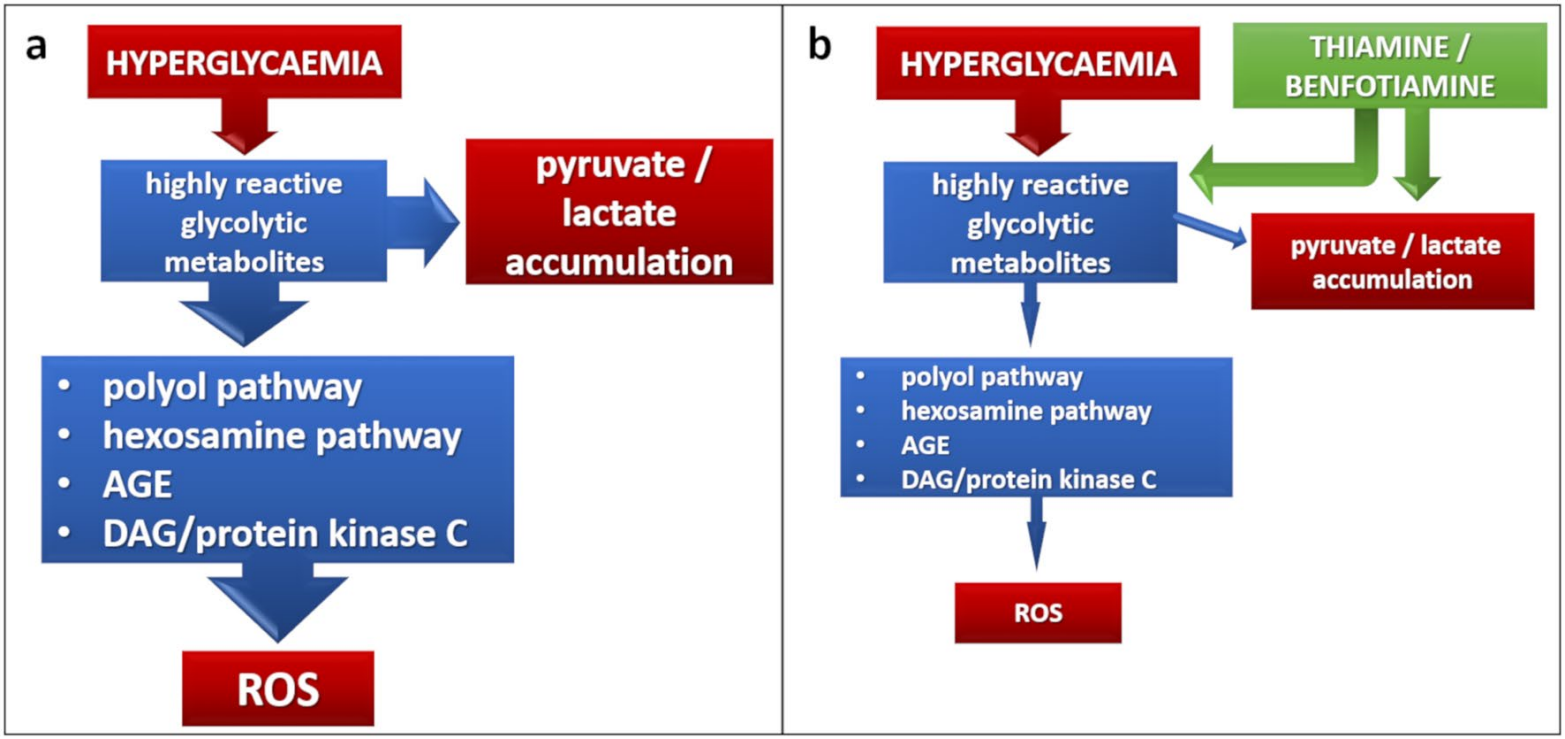
Mechanisms of action of thiamine as an antioxidant. **a** The “unifying mechanism” of ROS production according to Nishikawa et al. (2000): increased glycolytic flux due to excess glucose accelerates the four metabolic pathways of high-glucose damage: polyol pathway, hexosamine pathway, AGE production, and de novo synthesis of protein kinase C, leading to ROS accumulation. **b** Thiamine and benfotiamine normalize all these pathways in the presence of excess glucose, leading to reduced synthesis of ROS [56, 57]

The first evidence of the beneficial effects of thiamine on cells involved in diabetic microvascular complications is much more recent. In 1996, La Selva and co-workers demonstrated that thiamine neutralizes the damaging effects of high glucose concentrations in cultured endothelial cells, lowering lactate production and advanced glycation-end product (AGE) formation, and preserving cell replication [54]. Since then, several papers addressed the potential of the vitamin in counteracting diabetic microangiopathy. Actually, the possible key role of thiamine in accelerating glucose metabolism and eliminating toxic metabolites is intuitive. Cells involved in diabetic retinopathy and nephropathy are insulin-independent and, consequently, more prone to glucose-induced damage. Highly reactive intermediates of glycolysis accumulate into the cytoplasm and accelerate the four metabolic pathways involved in glucose damage: polyol pathway, hexosamine pathway, de novo synthesis of protein kinase C, and AGE formation, finally leading to the accumulation of reactive oxygen species (ROS) [55] (Fig. 2a). Thiamine and benfotiamine exert antioxidant properties, reducing enhanced ROS production in high glucose conditions by normalizing the four metabolic pathways described above [56, 57] (Fig. 2b). Thiamine addition also increases the oxidation of pyruvate to acetyl-CoA, thus counteracting the accumulation of pyruvate and lactate in the cytoplasm [58]. Excess accumulation of these metabolites due to thiamine deficiency may also increase hypoxia-inducible factor-1 *α* (HIF-1*α*) and, consequently, vascular endothelial growth factor (VEGF) expression, thus worsening diabetic retinopathy [59].

In addition to their antioxidant action, we demonstrated that thiamine and benfotiamine prevent cell damage and apoptosis induced by high glucose in retinal microvascular cells [8, 54, 57, 60, 61], and reduce glycation of basement membrane proteins, potentially preventing the detachment of pericytes from retinal capillary wall [62, 63]. In diabetic rats, benfotiamine prevented experimental retinopathy by inhibiting the formation of acellular capillaries [56]. Over-supplementation of thiamine and benfotiamine averted nephropathy in rats with streptozotocin-induced diabetes, inhibiting the development of microalbuminuria [64], dyslipidaemia, and advanced glycation of plasma proteins [65].

Dysregulation of thiamine transport inside cells may also play a role in thiamine deficiency and related disorders. High glucose induces downregulation of THTR1, THTR2 and their transcription factor Sp1, in human kidney proximal tubular epithelium [66]. Two single nucleotide polymorphisms located in the *SLC19A3* gene encoding for THTR2 may be associated with protection from diabetic retinopathy and nephropathy in type 1 diabetic subjects with long disease duration [67]. Subsequent work *in vitro* demonstrated that reduced thiamine availability concurs with high glucose in impairing thiamine transport inside both retinal and renal cells involved in diabetic microangiopathy, THTR2 being primarily involved [61, 68].

A few pilot studies in humans reported beneficial effects of thiamine administration on diabetic nephropathy. Type 2 diabetic patients with early stage nephropathy experienced reduction of urinary albumin excretion after three months of thiamine supplementation [69], while benfotiamine had fewer promising effects [70]. Despite all promising proofs-of-concept, the potential of thiamine in counteracting development or progression of retinopathy in human diabetes has not been addressed until now, possibly because of the necessity of running large, prolonged trials with sophisticated approaches to grade microangiopathy, especially retinal lesions.

## Old drugs and the impenetrable wall of evidence-based medicine

Over the last 80 years, a relevant amount of work *in vitro* and animal models has strongly suggested that thiamine supplementation may exert beneficial effects on the pathologic mechanisms of diabetes and its complications. Few studies in limited numbers of patients appear to confirm these observations. Most studies conclude by stressing the need for large structured clinical trials to test this vitamin as a potential and inexpensive option to prevent or slow down the progression of diabetes and its complications. However, these wishes have never materialized. One problem with the tenets of evidence-based medicine is that, apart from its undeniable advantages, it requires the completion of proper RCTs to gain registration for new indications. This approach is rightly reserved for new molecules, where the enormous investments for their development are justified by the prospect of higher returns. Although fully appropriate to leave old-fashioned practices behind and identify adverse effects, this effectively blocks the way to new indications for old medicines [71], including thiamine and its derivatives. An RCT to test their activity in diabetic complications would require the enrollment of thousands of patients, at least 4-year follow-up and sophisticated methods to centrally assess retinopathy, nephropathy, and neuropathy. As thiamine is non-patentable and inexpensive, it will be immensely difficult to raise the funds to run such trials. Even drugs that were subjected to proper RCTs may not make it: fenofibrate, a lipid-lowering agent, repeatedly reduced progression of retinopathy in a clinically significant way [72, 73] and candesartan, an angiotensin receptor blocker, prevented its onset [74, 75] but, as these were not pre-specified primary endpoints, neither gained the indication. The conundrum is that, in the general interest of evidence and safety, we may be depriving diabetic people of possible means to ameliorate their condition.

## Abbreviations

AGE: Advanced glycation-end products
HIF-1α: Hypoxia-inducible factor-1 α
TDP: Thiamine diphosphate
THTR1: Thiamine transporter-1
THTR2: Thiamine transporter-2
TK: Transketolase
TRMA: Thiamine-responsive megaloblastic anaemia
RCT: Randomized controlled clinical trial
ROS: Reactive oxygen species
VEGF: Vascular endothelial growth factor
